# Parental urinary biomarkers of preconception exposure to bisphenol A and phthalates in relation to birth outcomes

**DOI:** 10.1186/s12940-015-0060-5

**Published:** 2015-09-11

**Authors:** Melissa M. Smarr, Katherine L. Grantz, Rajeshwari Sundaram, José M. Maisog, Kurunthachalam Kannan, Germaine M. Buck Louis

**Affiliations:** 1 Division of Intramural Population Health Research, Eunice Kennedy Shriver National Institute of Child Health and Human Development, National Institutes of Health, 6100 Executive Blvd, Rockville, MD 20852 USA; 2 Division of Environmental Health Sciences, Wadsworth Center, New York State Department of Health and Department of Environmental Health Sciences, The University at Albany, Empire State Plaza, P.O. Box 509, Albany, NY USA

**Keywords:** Bisphenol A, Phthalates, Preconception exposure, Birth weight, Head circumference, Ponderal index, Endocrine disruptors

## Abstract

**Background:**

Bisphenol A (BPA) and phthalates are ubiquitous non-persistent endocrine disrupting chemicals whose relation with infant birth size is not clearly understood.

**Methods:**

We examined associations between maternal and paternal preconception urinary concentrations of total BPA and 14 phthalate metabolites and birth size for 233 infants. Multiple linear regression models were used to estimate parental quartiles of BPA and phthalates in relation to birth weight, length, head circumference, and ponderal index with separate models run for each parent adjusting for age, smoking, body mass index, education, alcohol, parity, and creatinine. Models also included an interaction term for each chemical and infant sex and were further adjusted to include the other partner’s chemical concentrations.

**Results:**

In maternal models adjusted for partner’s exposure and covariates, reductions in birth weight (range: 178-215 g; *p* < 0.05) were observed for the 2^nd^ quartile of maternal monomethyl phthalate, mono-[(2-carboxymethyl) hexyl] phthalate and mono-n-octyl phthalate when compared with the 1^st^ quartiles. The 3^rd^ quartile of monoethylhexyl phthalate (mEHP) was also associated with a 200.16 g (95 % CI: -386.90, -13.42) reduction. Similar reductions in birth weight were observed for the 2^nd^ quartile of paternal mEHP (β = -191.93 g; 95 % CI: -381.61, -2.25). Additionally, select maternal urinary metabolites were associated with decreased head circumference, birth length and gestational age. However, paternal concentrations were generally associated with increased birth length and gestational age.

**Conclusions:**

We observed some suggestion that preconception maternal and paternal urinary concentration of BPA and specific phthalate metabolites may be associated with smaller birth size and increased gestational age, though the findings appeared to be parent and chemical specific.

**Electronic supplementary material:**

The online version of this article (doi:10.1186/s12940-015-0060-5) contains supplementary material, which is available to authorized users.

## Background

Bisphenol A (BPA) and phthalates are non-persistent endocrine disrupting chemicals (EDCs) found in a variety of commercial products. Specifically, BPA is used in the production of polycarbonate plastics and epoxy resins, e.g., plastic bottles, lining of food cans, and thermal receipt papers [1]. Similarly, phthalates are plasticizers found in many consumer products, including but not limited to cosmetics, children’s toys, pharmaceuticals and nutritional supplements [2]. Biomonitoring data underscore their ubiquitous prevalence, with 93 % and 75 % of the U.S. general population having detectable concentrations of BPA and phthalates, respectively [3, 4]. In an analysis of urine samples collected on a subset of pregnant women from the 2003-2004 National Health and Nutritional Examination Survey (NHANES), BPA was detected in 96 % of the samples and phthalates were detected in 99-100 % of the samples [5]. Therefore, continual human exposure is probable, and the assessment of possible human health effects is essential.

Despite a suggestive body of animal evidence, current reviews conclude that studies on BPA and human health are limited [6–8]. An evolving body of epidemiologic research suggests that BPA may adversely affect human fecundity as measured by diminished semen quality [9], early blastocyst or embryonic development [10] and reduced levels of estradiol and number of oocytes retrieved [11] among women undergoing assisted reproductive technologies. Regarding pregnancy outcomes, maternal exposures have been associated with increased odds of preterm birth [12], reductions in length of gestation [13–15], birth weight, length [16] and fetal growth [17], suggesting a relation between parental exposures and fetal development.

To date, research has largely relied upon prenatal urinary concentrations and associated fetal/infant outcomes. To our knowledge, there have been no previous efforts focusing on parental preconception exposures to BPA and phthalates, despite the couple dependent nature of pregnancy. Such a study is important for helping to more fully understand the implications of physiological changes during pregnancy that may be associated with the reported variability of chemical exposures during this sensitive window of human development [18]. As such, preconception concentrations may be more reflective of early pregnancy concentrations that characterize women before they are typically recruited into pregnancy cohort studies. To date, few studies have focused on parental preconception or early pregnancy exposures other than a few focusing on persistent chemicals reported to be associated with infant birth size outcomes [19–21]. Other authors have noted the importance of considering paternal exposures given their ability to either directly impact sperm DNA or indirectly through seminal fluid exposure [22]. Prompted by these data gaps, we undertook an investigation of parental preconception BPA and phthalate exposures in relation to prospectively measured gestation and birth size, including the joint analysis of parental exposures.

## Materials and methods

### Study design and subjects

The study population comprises 501 reproductive aged couples who were recruited from16 counties in Michigan and Texas between 2005 and 2009 with the explicit purpose of assessing environmental influences on human fecundity and fertility, as previously described [23]. Couples were recruited upon discontinuing contraception for purposes of becoming pregnant. Inclusion criteria were minimal: females aged 18-40 and males ≥18 years; in a committed relationship; no physician diagnosis of infertility/sterility; females had to have menstrual cycles between 21-42 days without any injectable hormonal contraceptives in the past year; and an ability to communicate in English or Spanish. In-person interviews were conducted with each partner of the couple to ascertain lifestyle and reproductive history followed by standard anthropometric assessments to assess body mass index (BMI) [24]. Couples were followed for up to a year while trying for pregnancy, and women used home pregnancy test kits on the day of expected menstruation for the detection of pregnancy.

Among the 501 enrolled couples, 347 (69 %) had an observed human chorionic gonadotropin confirmed pregnancy of which 90 % occurred within 6 menstrual cycles and the remaining 10 % in cycles 7-12 [25]. Furthermore, 233 (67 %) had a singleton live birth, with a reported birth weight (exclusions for the present analysis included 2 couples with twin live births and 1 couple with a twin gestation and a singleton live birth). Pregnant women were followed daily through six post-conception weeks, then monthly until either a pregnancy loss or delivery. Institutional review board approvals were obtained from all collaborating institutions; couples gave written informed consent prior to study participation.

### Biospecimens collection and exposure assessment

During the enrollment home visit, research assistants instructed couples in the proper collection of urine samples for the quantification of BPA, phthalates, and creatinine. Samples were transported on ice for processing and shipment to the laboratory. Chemical analysis was performed at the Wadsworth Center, New York State Department of Health using an established protocol [26]. Specifically, urinary concentrations of total BPA were quantified as nanograms/milliliter (ng/mL) by enzymatic deconjugation, solid phase extraction and high-performance liquid chromatography (HPLC), coupled with API 2000 electrospray triple-quadrupole mass spectrometry (MS/MS). Urinalysis was performed using HPLC-MS/MS and an established protocol [27] for 14 phthalate metabolites: monomethyl phthalate (mMP), monoethyl phthalate (mEP), mono-n-butyl phthalate (mBP), mono (2-isobutyl phthalate) (miBP), monobenzyl phthalate (mBzP), mono (2-ethyl-5-carboxyphentyl) phthalate (mECPP), mono-[(2-carboxymethyl) hexyl] phthalate (mCMHP), mono (2-ethyl-5-oxohexyl) phthalate (mEOHP), mono (2-ethyl-5-hydroxyhexyl) phthalate (mEHHP), mono (2-ethylhexyl) phthalate (mEHP), mono (3-carboxypropyl) phthalate (mCPP), monocyclohexyl phthalate (mCHP), mono-isononyl phthalate (mNP), and monooctyl phthalate (mOP). Creatinine was quantified using a Roche/Hitachi Model 912 clinical analyzer (Dallas, TX) and the Creatinine Plus Assay. In addition, non-fasting blood samples were collected from each partner to measure serum cotinine using liquid chromatography-isotope dilution tandem mass spectrometry and as reported in ng/mL [28].

### Assessment of birth size

Following delivery, couples returned standardized birth announcements that were included in the pregnancy journals women completed while pregnant. This data collection form was designed for parents to report date-of-delivery, infant sex, birth weight in pounds and ounces (*n* = 233); length in -inches (*n* = 230); head circumference in inches (*n* = 183); and date of delivery (*n* = 233). Weight was converted to grams, and length and head circumference to centimeters for analysis. Gestational age was defined as the number of days from the day of ovulation as recorded on fertility monitors that was estimated to be the day of conception and date of delivery. We also estimated ponderal indices (PIs) for 230 infants, which is similar to adult body mass index and derived from the following formula: [birth weight (grams)/birth length (cm^3^)] × 100. PIs are proxies of infant adiposity and can be further categorized in relation to growth symmetry: normal (2.20-3.00), symmetrical (>3.00) and asymmetrical (<2.20) [29].

### Statistical analysis

Univariate analyses were performed to assess all chemical distributions and birth size outcomes. Machine-read values for all chemicals were used to avoid biasing regression estimates [30, 31]. Serum cotinine and urinary creatinine concentrations were natural-log transformed (ln) to approach more normal distributions. Geometric means and corresponding 95 % confidence intervals were estimated on ln-transformed chemical concentrations. We modeled all chemical data in quartiles in light of the non-normal distributions, absence of known linear relations with birth size and to aid in the interpretation of findings. To minimize possible bias associated with excluding individuals with missing data while maintaining power, we imputed data for missing chemical exposures stemming from insufficient urine for quantification and relevant covariates (10 %) using data for the entire cohort and Markov Chain Monte Carlo methods, under an assumption of ‘missing at random’ [32, 33]. We defined statistical significance as a two-sided *p*-value < 0.05.

Multiple linear regression models were used to estimate the mean change in each birth outcome per quartile of urinary chemical concentrations, relative to the lowest quartile; birth outcomes were modeled separately for each chemical and parent. Models were adjusted *a priori* for creatinine (ng/mL), age (years), race/ethnicity, body mass index (weight in kg/height in m^2^), education, smoking (serum cotinine ng/mL), frequency of alcoholic beverage consumption, maternal parity (number of live births) conditioned on gravidity (number of pregnancies) and infant gender. Models were re-run to test for potential interactions between each chemical and infant sex in light of data suggesting possible gender susceptibilities [34, 35]. Finally, models were further adjusted for the other partner’s chemical concentration, smoking and creatinine in light of pregnancy being a couple-dependent outcome. Testing for trend was conducted based on the linear trends of the association between BPA and phthalate urinary biomarker concentrations across the four intervals defined by their quartiles and the various birth outcomes. We determined a significant linear trend based on a two-sided *p*-value < 0.05. All analyses were performed using SAS software (version 9.4; SAS Institute, Cary, NC).

## Results

The cohort comprised mostly non-Hispanic White men and women who were college educated. The mean female and male ages were 29.8 ± 3.7 and 31.5 ± 4.6 years, respectively (Table 1). Prevalence of smoking and alcohol use at baseline was higher among males than females (10 % vs 4 % and 87 % vs 78 %, respectively). The cohort was largely healthy, except the slightly higher prevalence of self-reported hypertension among males (8 % vs 3 %). However, histories of chronic infection and diabetes were < 1 % among couples.

**Table 1:** Socio-demographic comparison of partners with a singleton live birth (*n* = 233),^a^ LIFE Study

Characteristic	Mothers	Fathers
Age (years): mean ± SD^†^	29.8 ± 3.7	31.5 ± 4.6
Body mass index (kg/m2): mean ± SD^†^	26.4 ± 6.6	28.9 ± 4.7
Race/Ethnicity: n (%)		
Non-Hispanic White	194 (84.0)	197 (84.9)
Non-Hispanic Black	2 (0.9)	4 (1.7)
Hispanic	20 (8.7)	20 (8.6)
Other	15 (6.4)	11 (4.8)
Education: n (%)		
< High school	0 (0)	2 (0.9)
High school	9 (3.9)	5 (2.2)
College	222 (96.1)	224 (97.8)
Smoking status: n (%)^†^		
Active (cotinine ≥ 100 ng/ml)	11 (4.7)	24 (10.3)
Passive (cotinine < 100 ng/ml)	222 (95.3)	209 (89.7)
Alcohol use: n (%)^†^		
No	52 (22.3)	31 (13.3)
Yes	181 (77.7)	201 (86.7)
History of diabetes: n (%)		
No	232 (99.6)	232 (99.6)
Yes	1 (0.4)	1 (0.4)
History of hypertension: n (%)		
No	226 (97.0)	213 (91.8)
Yes	7 (3.0)	19 (8.2)
History of chronic infections: n (%)		
No	232 (99.6)	232 (99.6)
Yes	1 (0.4)	1 (0.4)

^a^Missing covariate data is not reflected in this table

^†^*p* < 0.05 from independent *t*-test for continuous characteristics or chi-square test for categorical characteristics

The geometric means and accompanying 95 % CIs for urinary BPA and phthalate metabolites for each partner are displayed in Table 2. Overall, concentrations of BPA and select phthalates (mEP, mEHP, mEHHP, mECPP, mCMHP and mCPP) were significantly higher for males than females (*p* < 0.05). Generally, the percentage of urine samples with chemical concentrations below the limit of detection (LOD) were uniform across parental sex, with the exception of two chemicals, mMP and mEHP, which were more readily detectible in male than female urine samples (Table 2). Additionally, we assessed the correlation coefficients for maternal and paternal urinary BPA concentrations (r = 0.25), and the range of correlation coefficients for the various phthalate metabolites were between -0.03 to r = 0.33.

**Table 2:** Geometric means (95 % CI) for maternal and paternal preconception urinary metabolite concentrations

	Maternal urinary concentrations (*n* = 213)				Paternal urinary concentrations (*n* = 211)			
Chemical (ng/mL)	LOD (ng/mL)	% < LOD	% Negative	Geometric mean (95 % CI)	% < LOD	% Negative	Geometric mean (95 % CI)	p^a^
Creatinine	3.5	4.3	4.3	62.53 (55.7-70.2)	4.7	4.7	116 (106-128)	<0.001
BPA	0.02	11	9.4	0.38 (0.31-0.45)	11	11	0.59 (0.49-0.71)	0.00
*Low molecular weight*								
mMP	1.0	74	34	0.57 (0.44-0.76)	67	34	0.82 (0.64-1.07)	0.11
mEP	0.2	9.4	9.4	62.6 (50.3-77.9)	10	10	98.5 (78.4-124)	0.01
mBP	0.2	9.8	9.4	3.29 (2.75-3.94)	11	10	6.87 (5.76-8.19)	0.49
miBP	0.2	12	11	6.13 (5.15-7.29)	12	10	3.85 (3.27-4.54)	0.10
*DEHP metabolites*								
mEHP	1.0	64	60	3.92 (2.90-5.31)	54	49	4.54 (3.39-6.08)	0.02
mEHHP	0.2	11	9.8	9.18 (7.56-11.1)	11	11	14.7 (11.8-18.2)	0.00
mEOHP	0.2	13	9.8	5.15 (4.13-6.42)	11	10	7.03 (5.67-8.70)	0.09
mECPP	0.2	11	9.4	13.4 (10.9-16.4)	10	10	18.9 (15.4-23.1)	0.02
mCMHP	0.2	9.8	9.4	9.38 (7.72-11.4)	11	11	18.9 (15.36-23.3)	0.00
*High molecular weight*								
mBzP	0.2	13	9.8	2.90 (2.37-3.54)	14	11	3.40 (2.82-4.09)	0.32
mCHP	0.2	96	51	0.01 (0.01-0.02)	96	49	0.01 (0.01-0.02)	0.67
mOP	0.5	97	69	0.06 (0.04-0.08)	97	71	0.07 (0.04-0.10)	0.51
mCPP	0.2	14	9.8	3.12 (2.52-3.86)	14	11	5.47 (4.47-6.70	0.00
mNP	0.5	96	57	0.07 (0.05-0.09)	95	50	0.08 (0.06-0.11)	0.09

*Abbreviations:* *BPA* bisphenol (A; *mMP* monomethyl phthalate, *mEP* monoethyl phthalate, *mBP* mono-n-butyl phthalate, *miBP* monoisobutyl phthalate, *mEHP* monoethylhexyl phthalate, *mEHHP* mono-(2-ethyl-5-hydroxyhexyl) phthalate, *mEOHP* mono-(2-ethyl-5-oxohexyl) phthalate, *mECPP* mono-(5-carboxy-2-ethylpentyl) phthalate, *mCMHP* mono-[(2-carboxymethyl)hexyl] phthalate, *mBzP* monobenzyl phthalate, *mCHP* monocyclohexyl phthalate, *mOP* mono-n-octyl phthalate, *mCPP* mono(3-carboxypropyl) phthalate, *mNP* monoisononyl phthalate

^a^Reported *p*-values are from Wilcoxon-Mann–Whitney tests comparing the geometric means of maternal and paternal urinary chemical concentrations

Birth outcomes were not statistically different by infant sex, with the exception of birth weight (see Additional file 1). Mean birth weight was 3313.7 ± 447.1 g among girls and 3445.0 ± 488.7 g among boys (*p* = 0.03). Although not statistically significant, the lower end of the range of gestational age at birth was higher among girls (173-290 days) compared with boy infants (155-296). Additional details are provided in Additional file 1.

### Maternal urinary chemicals and birth outcomes

Overall, maternal biomarkers of preconception exposures to non-persistent EDCs did not have a strong linear association with measures of birth size. Maternal preconception urinary levels of BPA were not associated with any birth size outcomes (see Additional file 2). However, specific urinary levels for 8 out of 14 phthalates were significantly associated with decrements in birth size outcomes; importantly, no associations were observed for 6 of the 14 phthalates (Table 3). Most notably, negative associations were observed between phthalate metabolites of low molecular weight maternal compounds and birth weight. In adjusted models including the sum of paternal chemicals, for the 2^nd^ versus 1^st^ quartile of urinary mMP mean birth weight was reduced by 177.6 g (95 % CI: -344.9, -10.3). Likewise, for mothers in the 2^nd^ versus 1^st^ quartile of mEP a 200.2 g (95 % CI: -386.9, -13.4) decrease in birth weight was observed for infants. Overall, no associations were seen for birth length with one exception. Specifically, when comparing mothers in the 2^nd^ and 3^rd^ quartiles versus the 1^st^ quartile of mMP urinary concentrations a reduction in birth length was observed (β = -1.45 cm; 95 % CI: -2.8, -0.1 and β = -1.57 cm; 95 % CI: -2.6, -0.5, respectively) (Table 3). See Additional file 2 for all associations between maternal urinary concentrations and birth size outcomes not achieving statistical significance

**Table 3:** Associations between quartiles of maternal urinary BPA and phthalate concentrations and mean change in birth outcomes

	BW (g)	BL (cm)	HC (cm)	PI (g/cm^3^)	GA (days)
Chemical quartiles (ng/mL)	β (95 % CI)	β (95 % CI)	β (95 % CI)	β (95 % CI)	β (95 % CI)
*Low molecular weight*					
mMP					
1st (≤ -0.004)	Ref.	Ref.	Ref.	Ref.	Ref.
2nd (-0.001 - 0.33)	−177.6 (-344.9, -10.3)*	−1.6 (-2.6, -0.5)**	−0.3 (-1.1, 0.6)	0.1 (-0.1, 0.2)	−5.5 (-10.0, -1.0)*
3rd (0.34 - 1.68)	−168.9 (-375.7, 37.9)	−1.5 (-2.8, -0.1)*	−0.4 (-1.3, 0.6)	0.1 (0.0, 0.3)	−3.3 (-9.0, 2.5)
4th (≥1.69)	−94.0 (-280.5, 92.4)	−1.0 (-2.1, 0.1)	−0.2 (-1.1, 0.8)	0.1 (-0.1, 0.2)	−4.2 (-9.7, 1.4)
p-trend	0.94	0.33	0.70	0.34	0.45
mEP					
1st (≤ 26.9)	Ref.	Ref.	Ref.	Ref.	Ref.
2nd (27.3 - 65.3)	−34.6 (-225.4, 156.2)	−0.1 (-1.1, 1.0)	−0.3 (-1.1, 0.5)	0.0 (-0.2, 0.2)	2.7 (-2.2, 7.5)
3rd (66.1 - 203)	−200.2 (-386.9, -13.4)*	−0.1 (-1.2, 0.9)	−1.4 (-2.3, -0.6)**	−0.1 (-0.3, 0.1)	−1.8 (-6.6, 2.9)
4th (≥ 203)	−72.1 (-287.5, 143.4)	−0.3 (-1.5, 0.9)	−0.6 (-1.6, 0.4)	0.0 (-0.2, 0.2)	0.6 (-4.9, 6.1)
p-trend	0.80	0.52	0.36	0.58	0.19
*DEHP Metabolites*					
mEHP					
1st (≤ -1.89)	Ref.	Ref.	Ref.	Ref.	Ref.
2nd (-1.88 - -0.000716)	63.0 (-150.4, 276.3)	0.2 (-1.0, 1.3)	0.4 (-0.5, 1.3)	0.0 (-0.1, 0.1)	1.4 (-3.8, 6.6)
3rd (0.0340 - 3.73)	139.0 (-84.4, 362.5)	0.4 (-0.6, 1.4)	0.4 (-0.1, 1.5)	0.1 (-0.1, 0.2)	5.7 (0.5, 10.8)*
4th (≥ 3.81)	138.5 (-70.7, 347.6)	0.4 (-0.8, 1.6)	−0.2 (-1.4, 1.0)	0.1 (-0.1, 0.2)	3.6 (-2.2, 9.5)
p-trend	0.09	0.37	0.81	0.20	0.10
mEOHP					
1st (≤ 2.02)	Ref.	Ref.	Ref.	Ref.	Ref.
2nd (2.03 - 6.14)	−83.0 (-261.7, 95.8)	−0.6 (-1.7, 0.5)	−0.4 (-1.3, 0.5)	0.1 (-0.1, 0.2)	−0.3 (-5.2, 4.6)
3rd (6.15 - 15.6)	−162.4 (-350.4, 25.7)	−0.5 (-1.7, 0.8)	−1.3 (-2.2, -0.4)**	0.0 (-0.2, 0.1)	1.5 (-3.8, 6.7)
4th (≥ 15.8)	41.8 (-169.8, 253.5)	−0.1 (-1.3, 1.2)	−1.0 (-2.0, 0.1)	0.1 (-0.1, 0.2)	0.7 (-5.0, 6.4)
p-trend	0.43	0.93	0.18	0.21	0.56
mECPP					
1st (≤ 5.70)	Ref.	Ref.	Ref.	Ref.	Ref.
2nd (5.71 - 14.96)	−154.4 (-331.2, 22.5)	−0.5 (-1.6, 0.5)	−0.9 (-1.8, -0.1)*	0.0 (-0.2, 0.2)	2.0 (-2.9, 6.9)
3rd (14.98 -36.3)	−13.4 (-200.3, 173.5)	−0.1 (-1.4, 1.2)	−0.8 (-1.7, 0.2)	0.0 (-0.2, 0.2)	1.1 (-4.5, 6.7)
4th (≥ 36.7)	39.7 (-178.7, 258.1)	0.2 (-1.2, 1.7)	−0.9 (-2.0, 0.2)	0.0 (-0.2, 0.2)	1.9 (-4.6, 8.4)
p-trend	0.40	0.74	0.20	0.53	0.47
mCMHP					
1st (≤ 4.21)	Ref.	Ref.	Ref.	Ref.	
2nd (4.22 - 11.3)	−201.7 (-372.7, -30.7)**	−1.1 (-2.2, 0.0)	−0.2 (-1.1, 0.7)	0.0 (-0.1, 0.1)	−1.4 (-6.8, 4.0)
3rd (11.4 - 30.0)	−70.0 (-256.6, 118.7)	−0.4 (-1.6, 0.8)	−0.2 (-1.2, 0.9)	0.0 (-0.1, 0.2)	2.1 (-4.9, 9.1)
4th (≥ 30.03)	44.2 (-196.7, 285.1)	−0.5 (-1.9, 1.0)	−0.8 (-2.0, 0.3)	0.1 (-0.1, 0.4)	1.3 (-6.2, 8.8)
p-trend	0.68	0.38	0.19	0.14	0.65
*High Molecular Weight*					
mOP					
1st (≤ -0.110)	Ref.	Ref.	Ref.	Ref.	Ref.
2nd (-0.111 - -0.0563)	−215.4 (-387.1, -43.7)**	−0.71 (-1.8, 0.4)	−0.11 (-1.1, 0.8)	0.0 (-0.2, 0.1)	5.2 (-9.9, -0.4)*
3rd (-0.0564 - 0.022)	−75.3 (-283.3, 132.6)	−0.3 (-1.4, 0.8)	−0.5 (-1.5, 0.5)	0.0 (-0.1, 0.2)	−2.5 (-7.7, 2.7)
4th (≥ 0.024)	−81.8 (-251.7, 88.0)	0.12 (-0.9, 1.1)	−0.74 (-1.7, 0.2)	−0.1 (-0.2, 0.1)	−4.9 (-10.0, 0.3)
p-trend	0.30	0.91	0.11	0.40	0.11
mNP					
1st (≤ -0.079)	Ref.	Ref.	Ref.	Ref.	
2nd (-0.0789 - -0.0006)	−87.4 (-258.8, 83.9)	−0.4 (-1.5, 0.6)	0.4 (-0.5, 1.3)	0.0 (-0.1, 0.2)	−2.9 (-7.5, 1.7)
3rd (-0.0005 - 0.067)	53.9 (-126.1, 233.9)	0.7 (-0.5, 1.8)	0.1 (-0.9, 1.0)	0.0 (-0.2, 0.1)	−4.0 (-9.0, 1.0)
4th (≥ 0.074)	−149.8 (-327.4, 27.8)	−0.1 (-1.2, 0.9)	−0.1 (-1.0, 0.8)	−0.1 (-0.2, 0.1)	−7.0 (-12.3, -1.6)**
p-trend	0.30	0.95	0.98	0.40	**0.02**

NOTE: Significant findings are in boldface

*Abbreviations*: *BW* birth weight (grams), *BL* birth length (centimeters), *HC* head circumference (centimeters), *PI* Ponderal Index (grams/centimeters^3^), *GA* gestational age (days), *mMP* monomethyl phthalate, *mEP* monoethyl phthalate, *mEHP* monoethylhexyl phthalate, *mEOHP* mono-(2-ethyl-5-oxohexyl) phthalate, *mECPP* mono-(5-carboxy-2-ethylpentyl) phthalate, *mCMHP* mono-[(2-carboxymethyl)hexyl] phthalate, *mOP* mono-n-octyl phthalate, *mNP* monoisononyl phthalate

Models were adjusted for creatinine (ng/mL), age (years), race/ethnicity, BMI (kg/m2), education, cotinine (ng/mL), alcohol, conditional parity, infant gender, chemical*gender, paternal chemicals

*p < 0.05 **p ≤ 0.01

Several phthalates were associated with reductions in head circumference. Infants whose mothers had concentrations in the 3^rd^ versus 1^st^ quartile for mEP and mEOHP had smaller head circumference (β = -1.4 cm; 95 % CI: -2.3, -0.6 and β = -1.3 cm; 95 % CI: -2.2, -0.4, respectively) (Table 3). Increasing maternal exposure to Di-2-ethylhexyl phthalate (DEHP), as measured by the monoester metabolite mECPP, was also associated with reduced head circumference (β = -0.93 cm; 95 % CI: -1.8,-0.06), when comparing women in the 2nd versus 1^st^ quartile. None of the chemicals were significantly associated with ponderal indices when modeling maternal concentrations, including when further adjusting for paternal concentrations.

Finally, maternal preconception urinary phthalate metabolites were predominantly negatively associated with gestational age at birth. The 2^nd^ quartile of low molecular weight metabolite mMP, previously described, was associated with a 5.5 day (95 % CI: -10.0, -1.0) shorter gestational age, compared with the 1^st^ quartile (Table 3). Contrarily, when comparing infants whose mothers were in the 3^rd^ versus 1^st^ quartile of mEHP, a longer gestational age (β = 5.7 days; 95 % CI: 0.5, 10.8) was observed. With regard to high molecular weight phthalate metabolites, the 2^nd^ versus 1^st^ quartile of mOP was negatively associated with gestational age (β = -5.2 days; 95 % CI: - 9.9, -0.4). Additionally, comparing the 4^th^ and 1^st^ quartiles of maternal mNP reflected a significant 7- day (95 % CI: -12.3, -1.6) reduction in gestational age. A significant (*p* = 0.02) trend was observed between increasing quartiles of mNP and mean change in gestational age (Table 3).

### Paternal urinary chemicals and birth outcomes

With regard to paternal urinary phthalate concentrations, few negative associations were observed in the adjusted analysis, though different chemicals emerged. Paternal 2^nd^ quartile of mEHP concentrations were associated with reduced birth weight when compared with the 1^st^ quartile (β = -191.9 g; 95 % CI: -381.6, -2.3) (Table 4). Conversely, comparing the highest and the lowest quartiles of paternal mCHP, a positive association was observed with birth weight (β = 224.5 g; 95 % CI: 33.9, 415.0). An overall significant (*p* = 0.02) trend was observed between increasing paternal quartiles of urinary mCHP and mean change in birth weight.

**Table 4:** Associations between quartiles of paternal urinary BPA and phthalate concentrations and mean change in birth outcomes

	BW (g)	BL (cm)	HC (cm)	PI (g/cm^3^)	GA (days)
Chemical quartiles (ng/mL range)	β (95 % CI)	β (95 % CI)	β (95 % CI)	β (95 % CI)	β (95 % CI)
BPA					
1st (≤ 0.231)	Ref.	Ref.	Ref.	Ref.	Ref.
2nd (0.234 - 0.501)	102.00 (-93.35, 297.96)	0.80 (-0.24, 1.84)	0.59 (-0.33, 1.50)	−0.07 (-0.20, 0.07)	2.34 (-3.50, 8.18)
3rd (0.511- 1.13)	85.00 (-105.69, 276.23)	0.95 (-0.13, 2.03)	0.30 (-0.62, 1.22)	−0.10 (-0.23, 0.03)	2.48 (-3.20, 8.15)
4th (≥ 1.14)	178.53 (-14.88, 371.94)	1.35 (0.25, 2.45)*	0.44 (-0.45, 1.33)	−0.09 (-0.26, 0.07)	2.44 (-4.01, 8.90)
p-trend	0.07	**0.02**	0.34	0.25	0.44
*DEHP Metabolites*					
mEHP					
1st (≤ -1.44)	Ref.	Ref.	Ref.	Ref.	Ref.
2nd (-1.38 - 1.23)	−191.93 (-381.61, -2.25)*	−0.59 (-1.62, 0.45)	−0.76 (-1.64, 0.11)	−0.08 (-0.21, 0.06)	0.64 (-4.18, 5.46)
3rd (1.253 - 5.26)	−130.56 (-304.25, 43.13)	−0.11 (-1.06, 0.84)	−0.38 (-1.19, 0.43)	−0.08 (-0.19, 0.04)	3.29 (-1.31, 7.9)
4th (≥ 5.42)	1.61 (-176.84, 180.06)	−0.28 (-1.36, 0.8)	−0.35 (-1.23, 0.52)	0.03 (-0.1, 0.16)	0.92 (-4.35, 6.18)
p-trend	0.99	0.61	0.43	0.61	0.73
mEOHP					
1st (≤ 3.04)	Ref.	Ref.	Ref.	Ref.	Ref.
2nd (3.06 - 6.93)	−66.65 (-264.08, 130.78)	−0.14 (-1.39, 1.1)	−0.45 (-1.45, 0.55)	−0.02 (-0.18, 0.13)	7.23 (2.36, 12.1)**
3rd (6.94 - 17.7)	−48.23 (-266.56, 170.09)	−0.09 (-1.41, 1.22)	−0.55 (-1.49, 0.4)	−0.02 (-0.19, 0.14)	5.13 (0.02, 10.25)*
4th (≥ 17.8)	−65.30 (-317.61, 187.01)	−0.58 (-2.04, 0.88)	−0.56 (-1.56, 0.44)	0.05 (-0.1, 0.2)	3.22 (-1.96, 8.4)
p-trend	0.60	0.42	0.27	0.51	0.22
mECPP					
1st (≤ 8.53)	Ref.	Ref.	Ref.	Ref.	Ref.
2nd (8.60 - 20.2)	−9.91 (-224.03, 204.22)	−0.33 (-1.83, 1.17)	−0.37 (-1.29, 0.55)	0.06 (-0.15, 0.27)	7.33 (1.95, 12.71)**
3rd (20.3 - 46.2)	−13.26 (-217.02, 190.49)	−0.18 (-1.55, 1.19)	−0.15 (-1.18, 0.89)	0.00 (-0.22, 0.21)	5.08 (-0.57, 10.74)
4th (≥ 46.3)	7.90 (-235.19, 250.98)	−0.77 (-2.56, 1.03)	−0.35 (-1.4, 0.69)	0.14 (-0.07, 0.35)	6.38 (0.26, 12.5)*
p-trend	0.95	0.37	0.51	0.18	**0.04**
*High Molecular Weight*					
mCHP					
1st (≤ -0.0061)	Ref.	Ref.	Ref.	Ref.	Ref.
2nd (-0.006 - 0.003)	45.51 (-130.2, 221.23)	0.59 (-0.42, 1.59)	−0.04 (-0.88, 0.8)	−0.06 (-0.18, 0.06)	3.22 (-1.34, 7.78)
3rd (0.0026 - 0.0145)	147.16 (-20.82, 315.14)	0.30 (-0.71, 1.31)	0.45 (-0.44, 1.35)	0.03 (-0.10, 0.15)	2.65 (-2.05, 7.34)
4th (≥ 0.0148)	224.45 (33.94, 414.96)*	0.92 (-0.13, 1.98)	−0.52 (-1.32, 0.29)	0.03 (-0.10, 0.16)	7.01 (2.16, 11.86)**
p-trend	**0.02**	0.08	0.33	0.65	**0.00**
mOP					
1st (≤ -0.115)	Ref.	Ref.	Ref.	Ref.	Ref.
2nd (-0.114 - -0.0489)	−173.71 (-347.54, 0.11)	−1.22 (-2.33, -0.12)*	−0.52 (-1.32, 0.29)	0.06 (-0.09, 0.2)	−1.08 (-5.82, 3.67)
3rd (-0.0475 - 0.028)	−67.62 (-257.73, 122.49)	0.21 (-0.89, 1.3)	0.26 (-0.62, 1.13)	−0.08 (-0.20, 0.04)	−4.07 (-9.04, 0.89)
4th (≥ 0.029)	−64.61 (-243.21, 113.99)	−0.54 (-1.6, 0.52)	0.17 (-0.77, 1.11)	0.00 (-0.13, 0.13)	−1.29 (-6.13, 3.56)
p-trend	0.48	0.32	0.72	0.97	0.60

NOTE: Significant findings are in boldface

*Abbreviations*: *BW* birth weight (grams), *BL* birth length (centimeters), *HC* head circumference (centimeters), *PI* Ponderal Index (grams/centimeters^3^), *GA* gestational age (days), *BPA* bisphenol (A), *mEHP* monoethylhexyl phthalate, *mEOHP* mono-(2-ethyl-5-oxohexyl) phthalate, *mECPP* mono-(5-carboxy-2-ethylpentyl) phthalate, *mCHP* monocyclohexyl phthalate, *mOP* mono-n-octyl phthalate

Models were adjusted for creatinine (ng/mL), age (years), race/ethnicity, BMI (kg/m2), education, cotinine (ng/mL), alcohol, conditional parity, infant gender, chemical*gender, maternal chemicals

*p < 0.05 **p ≤ 0.01

In contrast to the observations based upon maternal urinary concentrations, the highest quartile of paternal urinary BPA concentration was positively associated with birth length (β = 1.4 cm; 95 % CI: 0.3, 2.5) (Table 4). The overall trend also was observed to be significant (*p* = 0.02) between increasing quartiles of paternal BPA and mean change in birth length (Table 4). A reduction in birth length was observed only between the 2^nd^ versus 1^st^ paternal quartile of mOP (β = -1.2 cm; 95 % CI: -2.3, -0.1). No associations were observed between paternal urinary chemicals and either head circumference or ponderal index. However, urinary phthalate metabolites were positively associated with gestational age. Specifically, the 2^nd^ and 3^rd^ quartiles of paternal mEOHP relative to the 1^st^ quartile were associated with a longer gestational age (β = 7.2 days; 95 % CI: 2.4, 12.1 and β = 5.1 days; 95 % CI: 0.02, 10.3, respectively, p for trend = 0.22). Likewise, the 2^nd^ and 4^th^ quartiles of mECPP were associated with a longer gestational age (β = 7.3 days; 95 % CI: 2.0, 12.7 and β = 6.4 days; 95 % CI: 0.3, 12.5, respectively, p for trend = 0.04). Finally, the 4^th^ quartile of paternal urinary concentrations of high molecular weight metabolite mCHP was associated with a 7.0 day (95 % CI = 2.2, 11.9) increase in gestational age, compared with the lowest quartile; an overall trend was observed for mean change in gestational age with increasing urinary levels of mCHP (p for trend = 0.005). Findings for paternal chemical concentrations and birth outcomes not achieving significance are provided in Additional file 3.

## Discussion

In this analysis of couples’ preconception urinary concentrations of short-lived endocrine disrupting chemicals and infant birth outcomes, we found parental urinary biomarkers for BPA and phthalates to be predominantly associated with reductions in length of gestation and birth size in a non-linear manner. While many signals of association were observed for specific quartiles of urinary EDCs, an overall linear trend between increasing biomarker quartiles and mean change in birth outcomes was only observed for 4 of the 15 chemicals and no chemical was consistently associated with reduction irrespective of parent. Specifically, we observed an overall linear trend between quartiles of maternal mNP and gestational age. Additionally, a linear trend was observed between quartiles of paternal BPA and birth length, mECPP and gestational age, and mCHP with birth weight and gestational age. Furthermore, we found the 2^nd^ quartiles of maternal mMP and mOP to be associated with a 5 day shorter length of gestation and a 178-215 g lowered birth weight. Similarly, the 4^th^ quartile of paternal mCHP was associated with the longest increase in gestational age and the largest increase in birth weight; this pattern was not observed for every birth size and gestational age association. Even with such large reductions in birth weight, there was no hint of IUGR estimated by the Ponderal Index proxy.

Overall, urinary chemical concentrations in this study population were lower than those reported for other U.S. populations. For example, in our analysis cohort, preconception urine concentrations of BPA had a geometric mean of 0.38 ng/mL (95 % CI = 0.31 – 0.45) for females and 0.59 (95 % CI = 0.49 – 0.71) for male partners, whereas an analysis of urine samples from a subset of the National Health and Nutrition Examination Survey (NHANES) reported a geometric mean BPA of 1.74 (95 % CI = 1.55 – 1.95), 1.97 (95 % CI = 1.80-2.16), and 1.73 (95 % CI = 1.60-1.87) μg/L for females and 2.09 (95 % CI = 1.92 – 2.28), 2.20 (95 % CI = 2.01-2.41), and 1.94 (95 % CI = 1.82-2.07) μg/L for males, during the 2005-2006, 2007-2008, and 2009-2010 study periods, compared with our analysis cohort for which data was also collected during the 2005-2009 period of time [36].

We are currently unaware of any previously published work that has focused on preconception exposure to BPA and phthalates as it relates to birth size precluding a more complete interpretation of our findings. Our findings are also limited by reliance on a single spot urine collected at the time the couples began trying for pregnancy. However, despite the low-to-moderate intraclass correlation coefficients of EDCs (0.10-0.51) across pregnancy [37], a single spot urine has the ability to be predictive of exposure ranging from weeks to months [38, 39]. Therefore our preconception urine samples of BPA and phthalates may reflect chemical levels during conception and possibly the first few weeks of pregnancy; a well-established critical window of fetal growth and development. This assumption is further supported by the concept of exposures to these non-persistent chemicals being continual on a daily basis [40–42]. Still, reliance on a single spot urine, whether collected prior to conception or during pregnancy, may not adequate characterize fetal exposure if one assumes changes in routes of exposures during these sensitive windows at any time, or if the many physiological changes required to support and maintain pregnancy affect internal doses and exposure quantification. Ideally, repeated samples within and across sensitive windows should be a part of future research methods to enable empirical assessment of exposure misclassification among other related considerations.

Furthermore, our study is strengthened by our assessment of paternal contribution to birth outcomes. Few epidemiological studies have been centered on paternal and/or couples’ non-occupational exposure to environmental chemicals as it relates to birth outcomes-none of which investigated BPA or phthalates. Nevertheless, previous analyses of maternal and paternal preconception exposures to persistent chemicals such as polybrominated diphenyl ethers and polychlorinated biphenyls and observed similar reductions in birth weight (21 to 195 g) [19], as did trace elements such as cesium, tungsten and uranium for birth weight (1.11 to 1.30 cm) and length (1.07 to 1.22 cm) [43]. Additionally, large effect estimates of phthalates on birth weight (-177 to -215 g) resemble a smoking during pregnancy effect [44, 45].

Still, cautious interpretation of results is warranted, given the potential for competing risks since this analysis was conditioned on having a live birth and EDCs have also been reported to be associated with couples’ ability to conceive [46]. However, the potential change in effect estimates would be relatively small and adjusting for common causes of the outcomes and fetal loss can reduce negative bias [47]. We also acknowledge the potential for spurious findings, given the comparison of multiple outcomes and chemicals. However when considering type I error for this analysis, many of our findings were statistically significant at with *p*-values ≤0.01, which is a much smaller chance of falsely rejecting the null hypothesis of no mean change in birth outcomes per quartile increase of urinary chemical concentration, compared with the lowest quartile of preconception chemical. Imputation improved our precision, in that significance was lower in our initial analyses without imputation (data not shown). We also dichotomized chemicals (mCHP and mOP) with a high percentage of concentrations below the LOD at or below the 75^th^percentile, and observed similar results as when modeling machine observed concentrations suggesting possible low dose effects. Despite these added analytic steps, cautious interpretation is needed in the interpretation of our findings as the findings await corroboration in light of these and other study limitations such as a relatively small cohort of infants and unknown birth outcomes for couples exiting the study before delivery.

Furthermore, the presence of many low-level chemical associations (2^nd^ and 3^rd^ quartiles) with birth size may not simply be explained as the result of limited sample size or overestimating the effect of urinary biomarkers of exposure in light of exposure misclassification. A growing body of evidence suggests that the endocrine system is sensitive to low-dose effects of various chemicals. Furthermore, animal studies of EDCs that act as antiandrogens or estrogen have shown that low-dose effects is not only chemical specific but varies by endpoint as well and may have global dynamic effects on cells, tissues and organs [48–50].

While the biological explanation behind these findings is not clearly understood, early pregnancy maternal exposures to EDCs have been associated with oxidative damage in rats, sheep and humans [51], and oxidative stress has been shown to play a role in intrauterine growth restriction (IUGR) [52, 53], and measures of fetal anthropometry at birth have been established as proxies for the assessment of IUGR. A previous analysis of phthalates and oxidative stress among the present cohort observed an increase in oxidative stress biomarker 8-hydroxy-2′-deoxyguanosine, with increased urinary concentrations of select phthalate metabolites [54]. We also consider the role of paternal exposures [55] found male urinary concentrations of select phthalate metabolites to be associated with diminished fecundity as measured by a longer time-to-pregnancy (TTP), although female urinary phthalate exposures were not associated with a longer TTP. DNA methylation is also suspected to affect select growth genes as a result of first trimester maternal phthalate exposure [56]. Animal studies suggest that DNA methylation may play a role in short-lived EDCs transgenerational epigenetic effects, which result in adverse reproductive outcomes in later offspring generations [57, 58]. A recent study of male mice exposed to dioxin found that even when mated with non-exposed female mice, multiple subsequent generations presented with reduced fertility and spontaneous preterm birth [59]. Additionally, male offspring of mice in one study had increased spermatogenic cell apoptosis and differential DNA methylation regions in the offspring sperm were consistent with a specific exposure lineage back to the original exposure of the first generation male mouse [60].

## Conclusions

To our knowledge, this study represents an initial attempt to utilize a couple-based exposure design to assess the relation between maternal and paternal preconception urinary concentrations of BPA and phthalates and infant gestation and birth size. In the context of an observational design and exploratory analytic strategy, we observed some evidence that BPA and some but not all phthalates were associated with shorter gestations and smaller infants in relation to maternal exposure, but longer gestations and larger infants in relation to paternal exposures. Collectively, the findings underscore the importance of couple based designs for assessing the reproductive and/or developmental toxicity of environmental phenols and other endocrine disrupting chemicals.

## Additional files

Additional file 1: **Distribution of birth outcomes by infant sex, LIFE Study, 2005-2009.** (PDF 19 kb)
Additional file 2: **Effect estimates of maternal urinary BPA, phthalate quartiles and mean change in birth outcomes, with no observed statistical significance, LIFE Study, 2005-2009.** (DOCX 21 kb)
Additional file 3: **Effect estimates of paternal urinary BPA, phthalate quartiles and mean change in birth outcomes, with no observed statistical significance, LIFE Study, 2005-2009.** (DOCX 26 kb)

## Abbreviations

BMI: Body mass index
BPA: Bisphenol A
DNA: Deoxyribose nucleic acid
EDCs: Endocrine disrupting chemicals
IUGR: Intrauterine growth restriction
LOD: Limit of detection
mBP: Mono-n-butyl phthalate
mBzP: Monobenzyl phthalate
mCHP: Monocyclohexyl phthalate
mCMHP: Mono-[(2-carboxymethyl)hexyl] phthalate
mCPP: Mono(3-carboxypropyl) phthalate
mECPP: Mono-(5-carboxy-2-ethylpentyl) phthalate
mEHHP: Mono-(2-ethyl-5-hydroxyhexyl) phthalate
mEHP: Monoethylhexyl phthalate
mEOHP: Mono-(2-ethyl-5-oxohexyl) phthalate
mEP: Monoethyl phthalate
miBP: Monoisobutyl phthalate
mMP: Monomethyl phthalate
mNP: Monoisononyl phthalate
mOP: Mono-n-octyl phthalate
PI: Ponderal index
TTP: Time-to -pregnancy
