# Supplementary material for: Parental urinary biomarkers of preconception exposure to bisphenol A and phthalates in relation to birth outcomes
Source: Environ Health. 2015 Sep 11;14:73. doi: 10.1186/s12940-015-0060-5 (PMC4567813; doi:10.1186/s12940-015-0060-5)
Supplement: Additional file 1: — Distribution of birth outcomes by infant sex, LIFE Study, 2005-2009. (PDF 19 kb) [file 12940_2015_60_MOESM1_ESM.pdf]

Distribution of birth outcomes by infant sex, LIFE Study, 2005-2009.

	All		Girls		Boys	
**Birth Outcome**	**Mean (SD)**	**Min-Max**	**Mean (SD)**	**Min-Max**	**Mean (SD)**	**Min-Max**
Birth Weight (grams)‡	3379.9 (472.3)	2012.8 - 4762.7	3313.7 (447.1)	2126.2 - 4252.4	3445.0 (488.7)	2012.8 - 4762.7
Length (cm)	50.5 (2.7)	43.2 - 55.9	50.3 (2.7)	43.2 - 55.9	50.8 (2.6)	43.2 - 55.9
Head Circumference (cm)	34.8 (2.2)	27.9 - 48.3	34.5 (2.0)	30.5 - 40.6	34.9 (1.8)	27.9 -38.1
Ponderal Index (g/cm^3)	2.6 (0.3)	1.8 - 3.6	2.6 (0.3)	1.8 - 3.6	2.6 (0.3)	2.0 - 3.6
Gestational Age (days)	257.4 (12.4)	213.0 - 296.0	256.6 (15.6)	173.0 -290.0	256.3 (15.5)	155.0 -296.0

Abbreviations: N, sample size; SD, standard deviation; Min, minimum; Max, maximum.

[a]Number of participants with birth outcome data (N) for all: birth weight (233), length (230), head circumference (183), ponderal index (230), gestational age (200); (N) for girls (birth weight (116), length (115), head circumference (90), ponderal index (115), gestational age (98);); (N) for boys (birth weight (114), length (113), head circumference (90), ponderal index (113), gestational age (103);
