# Supplementary material for: Parental urinary biomarkers of preconception exposure to bisphenol A and phthalates in relation to birth outcomes
Source: Environ Health. 2015 Sep 11;14:73. doi: 10.1186/s12940-015-0060-5 (PMC4567813; doi:10.1186/s12940-015-0060-5)
Supplement: Additional file 2: — Effect estimates of maternal urinary BPA, phthalate quartiles and mean change in birth outcomes, with no observed statistical significance, LIFE Study, 2005-2009. (DOCX 21 kb) [file 12940_2015_60_MOESM2_ESM.docx]

Effect estimates of maternal urinary BPA, phthalate quartiles and mean change in birth outcomes, with no observed statistical significance, LIFE Study, 2005-2009.

|  | **BW (g)** | **BL (cm)** | **HC (cm)** | **PI (g/cm^3^)** | **GA (days)** |
| --- | --- | --- | --- | --- | --- |
| **Chemical Quartiles (ng/mL)** | **β (95% CI)** | **β (95% CI)** | **β (95% CI)** | **β (95% CI)** | **β (95% CI)** |
| BPA |  |  |  |  |  |
| 1st (≤ 0.169) | Ref. | Ref. | Ref. | Ref. | Ref. |
| 2nd (0.172 - 0.392) | 85.34 (-112.04, 282.72) | 0.72 (-0.29, 1.72) | 0.46 (-0.55, 1.46) | -0.02 (-0.15, 0.10) | 3.31 (-2.51, 9.13) |
| 3rd (0.393 - 0.94) | -150.50 (-350.91, 49.92) | -0.31 (-1.33, 0.72) | -0.88 (-1.83, 0.08) | -0.03 (-0.18, 0.11) | 2.13 (-2.76, 7.01) |
| 4th (≥ 0.97) | -55.65 (-261.21, 149.91) | 0.19 (-1.15, 1.54) | 0.02 (-0.99, 1.02) | -0.05 (-0.23, 0.14) | -0.16 (-5.26, 4.95) |
| p-trend | 0.59 | 0.77 | 0.97 | 0.60 | 0.95 |
| *Low Molecular Weight* |  |  |  |  |  |
| mBP |  |  |  |  |  |
| 1st (≤ 2.94) | Ref. | Ref. | Ref. | Ref. |  |
| 2nd (2.30 - 7.85) | -149.29 (-370.14, 71.57) | -0.13 (-1.38, 1.13) | -0.29 (-1.13, 0.55) | -0.10 (-0.23, 0.03) | -1.17 (-8.56, 6.22) |
| 3rd (7.89 - 16.85) | -107.62 (-313.02, 97.77) | 0.08 (-1.42, 1.58) | -0.30 (-1.33, 0.74) | -0.08 (-0.27, 0.11) | -1.97 (-8.82, 4.87) |
| 4th (≥ 16.86) | -88.56 (-367.25, 190.12) | 0.61 (-1.07, 2.30) | -0.98 (-2.46, 0.51) | -0.16 (-0.33, 0.00) | -1.92 (-11.4, 7.56) |
| p-trend | 0.94 | 0.51 | 0.35 | 0.23 | 0.90 |
| miBP |  |  |  |  |  |
| 1st (≤ 1.36) | Ref. | Ref. | Ref. | Ref. | Ref. |
| 2nd (1.37 - 3.99) | -54.63 (-228.51, 119.24) | -0.27 (-1.3, 0.76) | 0.22 (-0.66, 1.09) | -0.04 (-0.16, 0.08) | -2.01 (-6.8, 2.78) |
| 3rd (4.01 - 8.51) | 14.51 (-177.63, 206.66) | -0.24 (-1.34, 0.86) | -0.49 (-1.46, 0.48) | 0.04 (-0.1, 0.17) | 0.13 (-5.41, 5.66) |
| 4th (≥ 8.55) | -73.23 (-299.79, 153.33) | 0.03 (-1.46, 1.52) | -0.41 (-1.55, 0.73) | -0.06 (-0.25, 0.12) | -1.34 (-7.76, 5.08) |
| p-trend | 0.71 | 0.81 | 0.22 | 0.80 | 0.62 |
| *DEHP Metabolites* |  |  |  |  |  |
| mEHHP |  |  |  |  |  |
| 1st (≤ 4.12) | Ref. | Ref. | Ref. | Ref. |  |
| 2nd (4.16 - 10.77) | -88.10 (-272.33, 96.13) | -0.62 (-1.66, 0.41) | -0.42 (-1.31, 0.47) | 0.05 (-0.07, 0.17) | 0.18 (-4.81, 5.18) |
| 3rd (10.78 -25.8) | -61.33 (-271.6, 148.95) | -0.22 (-1.32, 0.87) | -0.68 (-1.63, 0.28) | 0.01 (-0.12, 0.14) | -2.05 (-7.32, 3.21) |
| 4th ( ≥ 25.9) | 2.15 (-214.99, 219.29) | -0.30 (-1.42, 0.82) | -0.70 (-1.82, 0.42) | 0.08 (-0.09, 0.25) | 1.69 (-3.87, 7.25) |
| p-trend | 0.64 | 0.56 | 0.43 | 0.19 | 0.58 |
| *High Molecular Weight* |  |  |  |  |  |
| mBzP |  |  |  |  |  |
| 1st (≤ 1.25) | Ref. | Ref. | Ref. | Ref. |  |
| 2nd (1.26 - 3.70) | 13.26 (-192.56, 219.08) | 0.00 (-0.99, 0.99) | 0.6 (-0.26, 1.45) | 0.04 (-0.09, 0.17) | 0.78 (-4.69, 6.26) |
| 3rd (3.72 - 9.02) | -55.17 (-253.01, 142.68) | -0.14 (-1.30, 1.03) | -0.47 (-1.43, 0.5) | -0.01 (-0.14, 0.12) | -2.94 (-9.28, 3.4) |
| 4th (≥9.10) | -98.51 (-323.83, 126.82) | -0.17 (-1.65, 1.31) | -0.33 (-1.41, 0.75) | -0.04 (-0.23, 0.15) | -2.44 (-10.07, 5.18) |
| p-trend | 0.71 | 0.89 | 0.43 | 0.98 | 0.60 |
| mCHP |  |  |  |  |  |
| 1st (≤ -0.0079) | Ref. | Ref. | Ref. | Ref. | Ref. |
| 2nd (-0.0077 - 0.0017) | -67.07 (-262.38, 128.25) | -0.20 (-1.19, 0.79) | -0.41 (-1.25, 0.43) | -0.02 (-0.16, 0.11) | 0.28 (-4.64, 5.21) |
| 3rd (0.0018 - 0.0141) | -36.46 (-219.19, 146.27) | 0.36 (-0.65, 1.38) | 0.09 (-0.76, 0.93) | -0.11 (-0.23, 0.01) | -0.48 (-5.69, 4.72) |
| 4th (≥ 0.0145) | -186.5 (-383.17, 10.16) | -0.36 (-1.37, 0.65) | -0.3 (-1.23, 0.64) | -0.08 (-0.22, 0.06) | -0.85 (-6.42, 4.72) |
| p-trend | 0.13 | 0.59 | 0.24 | 0.34 | 0.62 |
| mCPP |  |  |  |  |  |
| 1st (≤ 1.29) | Ref. | Ref. | Ref. | Ref. |  |
| 2nd (1.31 - 3.95) | -12.17 (-193.95, 169.6) | -0.72 (-1.73, 0.29) | 0.6 (-0.22, 1.41) | 0.09 (-0.06, 0.23) | 0.35 (-4.21, 4.91) |
| 3rd (4.01 - 9.78) | 10.08 (-185.02, 205.18) | -0.10 (-1.7, 1.51) | 0.23 (-0.79, 1.25) | 0.04 (-0.17, 0.24) | 2.98 (-3.62, 9.58) |
| 4th (≥ 9.93) | -68.67 (-337.76, 200.41) | -0.22 (-1.99, 1.55) | 0.20 (-1.04, 1.44) | 0.00 (-0.23, 0.23) | 0.92 (-7.18, 9.01) |
| p-trend | 0.80 | 0.68 | 0.89 | 0.70 | 0.49 |

Abbreviations: BW, birth weight (grams); BL, birth length (centimeters); HC, head circumference (centimeters); PI, Ponderal Index (grams/centimeters^3^); GA, gestational age (days); BPA, bisphenol (A); mBP, mono-n-butyl phthalate; miBP, monoisobutyl phthalate; mEHHP, mono-(2-ethyl-5-hydroxyhexyl) phthalate; mBzP, monobenzyl phthalate; mCHP, monocyclohexyl phthalate; mCPP, mono(3-carboxypropyl) phthalate. Models were adjusted for creatinine (ng/mL), age (years), race/ethnicity, BMI (kg/m2), education, cotinine (ng/mL), alcohol, conditional parity, infant gender, chemical*gender, paternal chemicals.
