# Supplementary material for: Parental urinary biomarkers of preconception exposure to bisphenol A and phthalates in relation to birth outcomes
Source: Environ Health. 2015 Sep 11;14:73. doi: 10.1186/s12940-015-0060-5 (PMC4567813; doi:10.1186/s12940-015-0060-5)
Supplement: Additional file 3: — Effect estimates of paternal urinary BPA, phthalate quartiles and mean change in birth outcomes, with no observed statistical significance, LIFE Study, 2005-2009. (DOCX 26 kb) [file 12940_2015_60_MOESM3_ESM.docx]

Effect estimates of paternal urinary BPA, phthalate quartiles and mean change in birth outcomes, with no observed statistical significance, LIFE Study, 2005-2009.

|  | **BW (g)** | **BL (cm)** | **HC (cm)** | **PI (g/cm^3^)** | **GA (days)** |
| --- | --- | --- | --- | --- | --- |
| Chemical Quartiles (ng/mL) | **β (95% CI)** | **β (95% CI)** | **β (95% CI)** | **β (95% CI)** | **β (95% CI)** |
| *Low Molecular Weight* |  |  |  |  |  |
| mMP |  |  |  |  |  |
| 1st (≤ 0.0302) | Ref. | Ref. | Ref. | Ref. | Ref. |
| 2nd (0.0309 - 0.568) | 2.7 (-176.3, 181.7) | 0.4 (-0.7, 1.4) | -0.1 (-1.0, 0.8) | -0.1 (-0.2, 0.1) | 4.6 (-0.1, 9.3) |
| 3rd (0.573 - 2.01) | -8.7 (-200.6, 184.3) | -0.2 (-1.2, 0.9) | -0.6 (-1.5, 0.4) | 0.0 (-0.1, 0.1) | 3.6 (-1.1, 8.3) |
| 4th (≥ 2.02) | 92.6(-105.2, 290.3) | -0.4 (-1.5, 0.7) | -0.1 (-1.0, 0.9) | 0.1 (-0.1, 0.3) | -0.2 (-5.1, 4.8) |
| p-trend | 0.36 | 0.48 | 0.88 | 0.19 | 0.94 |
| mEP |  |  |  |  |  |
| 1st (≤ 31.7) | Ref. | Ref. | Ref. | Ref. | Ref. |
| 2nd (32.1 - 87.2) | -159.5 (-354.5, 35.5) | -0.9 (-1.9, 0.1) | -0.5 (-1.5, 0.4) | 0.0 (-0.2, 0.1) | -3.3 (-8.1, 1.5) |
| 3rd (87.4 - 275) | -31.3 (-247.8, 185.3) | -0.2 (-1.5, 1.1) | -0.9 (-1.8, 0.1) | 0.0 (-0.2, 0.1) | 1.2 (-4.0, 6.4) |
| 4th (≥ 277) | -87.2 (-321.0, 146.6) | -0.8 (-2.2, 0.6) | -0.1 (-1.1, 0.8) | 0.0 (-0.1, 0.2) | -2.6 (-8.0, 2.9) |
| p-trend | 0.45 | 0.24 | 0.77 | 0.52 | 0.35 |
| mBP |  |  |  |  |  |
| 1st (≤ 3.30) | Ref. | Ref. | Ref. | Ref. | Ref. |
| 2nd (3.35- 7.39) | 56.2 (-134.0, 246.3) | 0.4 (-0.7, 1.5) | -0.1 (-1.1, 0.9) | 0.0 (-0.2, 0.2) | -0.9 (-6.1, 4.4) |
| 3rd (7.40 - 14.8) | 131.2 (-82.8, 345.2) | 0.5 (-0.8, 1.7) | -0.4 (-1.5, 0.6) | 0.0 (-0.2, 0.2) | 2.3 (-3.5, 8.0) |
| 4th (≥ 14.9) | 63.6 (-168.0, 295.1) | 0.6 (-0.7, 1.9) | -0.4 (-1.6, 0.8) | 0.0 (-0.2, 0.2) | 1.1 (-5.5, 7.8) |
| p-trend | 0.59 | 0.39 | 0.50 | 0.72 | 0.73 |
| miBP |  |  |  |  |  |
| 1st (≤ 1.82) | Ref. | Ref. | Ref. | Ref. | Ref. |
| 2nd (1.83 - 4.37) | 91.2 (-93.0, 275.5) | -0.1 (-1.2, 1.0) | -0.2 (-1.1, 0.7) | 0.1 (-0.1, 0.2) | 3.9 (-1.2, 9.1) |
| 3rd (4.45 - 9.08) | 135.7 (-100.4, 371.8) | 0.0 (-1.5, 1.6) | -0.2 (-1.3, 0.8) | 0.1 (-0.1, 0.3) | 1.6 (-4.1, 7.3) |
| 4th (≥ 9.10) | 131.4 (-122.0, 384.8) | 0.6 (-1.1, 2.3) | 0.2 (-1.0, 1.3) | 0.0 (-0.2, 0.2) | 1.7 (-4.5, 7.8) |
| p-trend | 0.30 | 0.46 | 0.77 | 0.95 | 0.60 |
| *DEHP Metabolites* |  |  |  |  |  |
| mEHHP |  |  |  |  |  |
| 1st (≤ 5.54) | Ref. | Ref. | Ref. | Ref. | Ref. |
| 2nd (5.56 - 14.3) | -147.5 (-330.7, 35.8) | -0.4 (-1.5, 0.8) | -0.5 (-1.4, 0.4) | 0.0 (-0.2, 0.1) | 4.0 (-1.4, 9.3) |
| 3rd (14.7 - 37.8) | 31.1 (-150.4, 212.7) | -0.1(-1.3, 1.1) | -0.4 (-1.3, 0.5) | 0.0 (-0.1, 0.2) | 4.2 (-0.7, 9.1) |
| 4th (≥ 37.9) | -25.5 (-215.0, 164.0) | -0.4 (-1.7, 0.8) | -0.5 (-1.5, 0.5) | 0.1 (-0.1, 0.3) | 1.5 (-3.8, 6.8) |
| p-trend | 0.79 | 0.50 | 0.31 | 0.61 | 0.58 |
| mCMHP |  |  |  |  |  |
| 1st (≤ 6.61) | Ref. | Ref. | Ref. | Ref. | Ref. |
| 2nd (6.68 - 18.4) | -30.4 (-225.5, 164.8) | -0.3 (-1.6, 1.0) | -0.4 (-1.5, 0.7) | 0.0 (-0.2, 0.2) | 1.0 (-4.5, 6.4) |
| 3rd (18.5 - 47.2) | 28.6 (-223.3, 280.5) | 0.1 (-1.2, 1.3) | 0.2 (-0.9, 1.2) | 0.0 (-0.2, 0.2) | 3.1 (-2.7, 8.9) |
| 4th (≥ 46.74) | 26.4 (-222.4, 275.1) | -0.5 (-1.9, 0.9) | -0.5 (-1.7, 0.7) | 0.1 (-0.1, 0.3) | 2.2 (-3.7, 8.1) |
| p-trend | 0.83 | 0.50 | 0.40 | 0.31 | 0.47 |
| *High Molecular Weight* |  |  |  |  |  |
| mBzP | *  * | | |  |  |
| 1st (≤ 1.52 ) | Ref. | Ref. | Ref. | Ref. | Ref. |
| 2nd (1.56 - 3.70) | -174.1 (-397.2, 49.1) | -0.7 (-1.9, 0.5) | -0.2 (-1.2, 0.9) | 0.0 (-0.2, 0.1) | -3.1 (-8.7, 2.6) |
| 3rd (3.71 - 8.52) | -111.4 (-353.4, 130.6) | -0.4 (-1.8, 1.1) | -0.1 (-1.0, 0.9) | 0.0 (-0.2, 0.2) | -4.0 (-10.8, 2.8) |
| 4th (≥ 8.59) | -87.0 (-370.5, 196.5) | 0.3 (-1.3, 1.9) | -0.1 (-1.4, 1.2) | -0.2 (-0.4, 0.1) | -2.6 (-10.2, 5.0) |
| p-trend | 0.54 | 0.71 | 0.89 | 0.15 | 0.49 |
| mCPP |  |  |  |  |  |
| 1st (≤2.46) | Ref. | Ref. | Ref. | Ref. | Ref. |
| 2nd (2.50 - 5.57) | -58.2 (-314.1, 197.7) | 0.2 (-1.2, 1.5) | -0.2 (-1.1, 0.8) | -0.1 (-0.2, 0.0) | 2.0 (-3.5, 7.4) |
| 3rd (5.58 - 12.13) | 0.5 (-269.4, 270.3) | -0.4 (-1.9, 1.1) | 0.1 (-0.9, 1.1) | 0.1 (-0.1, 0.2) | -1.29 (-6.6, 4.0) |
| 4th (≥ 12.17) | 2.3 (-291.6, 296.2) | 0.4 (-1.2, 1.9) | 0.3 (-0.8, 1.3) | -0.1 (-0.2, 0.1) | 3.4 (-2.6, 9.3) |
| p-trend | 0.99 | 0.64 | 0.61 | 0.26 | 0.26 |
| mNP |  |  |  |  |  |
| 1st (≤ -0.0699) | Ref. | Ref. | Ref. | Ref. | Ref. |
| 2nd (-0.0672 - 0.0052) | 0.7 (-183.6, 185.0) | -0.4 (-1.6, 0.7) | 0.2 (-0.7, 1.2) | 0.1 (0.0, 0.2) | -3.0 (-8.0, 2.0) |
| 3rd (0.0053 - 0.083) | -57.4 (-245.9, 131.0) | -0.7 (-2.0, 0.5) | 0.0 (-0.9, 0.9) | 0.1 (0.0, 0.2) | -2.0 (-7.0, 3.1) |
| 4th (≥ 0.086) | 25.5 (-150.5, 201.5) | 0.1 (-1.1, 1.2) | 0.7 (-0.2, 1.6) | 0.1 (-0.1, 0.2) | -0.8 (-5.9, 4.4) |
| p-trend | 0.78 | 0.90 | 0.12 | 0.46 | 0.77 |

Abbreviations: BW, birth weight (grams); BL, birth length (centimeters); HC, head circumference (centimeters); PI, Ponderal Index (grams/centimeters^3^); GA, gestational age (days); mMP, monomethyl phthalate; mEP, monoethyl phthalate; mBP, mono-n-butyl phthalate; miBP, monoisobutyl phthalate; mEHHP, mono-(2-ethyl-5-hydroxyhexyl) phthalate; mCMHP, mono-[(2-carboxymethyl)hexyl] phthalate; mBzP, monobenzyl phthalate; mCPP, mono(3-carboxypropyl) phthalate; mNP, monoisononyl phthalate.

Models were adjusted for creatinine (ng/mL), age (years), race/ethnicity, BMI (kg/m2), education, cotinine (ng/mL), alcohol, conditional parity, infant gender, chemical*gender, maternal chemicals.
